# Global Functional Analyses of Cellular Responses to Pore-Forming Toxins

**DOI:** 10.1371/journal.ppat.1001314

**Published:** 2011-03-03

**Authors:** Cheng-Yuan Kao, Ferdinand C. O. Los, Danielle L. Huffman, Shinichiro Wachi, Nicole Kloft, Matthias Husmann, Valbona Karabrahimi, Jean-Louis Schwartz, Audrey Bellier, Christine Ha, Youn Sagong, Hui Fan, Partho Ghosh, Mindy Hsieh, Chih-Shen Hsu, Li Chen, Raffi V. Aroian

**Affiliations:** 1 Section of Cell and Developmental Biology, University of California, San Diego, La Jolla, California, United States of America; 2 Center for Eukaryotic Gene Regulation, Department of Biochemistry and Molecular Biology, Pennsylvania State University, University Park, Pennsylvania, United States of America; 3 Institute of Medical Microbiology and Hygiene, University Medical Center, Johannes Gutenberg-University Mainz, Hochhaus am Augustusplatz, Mainz, Germany; 4 Groupe d'étude des protéines membranaires, Université de Montréal, Montreal, Quebec, Canada; 5 Department of Chemistry and Biochemistry, University of California, San Diego, La Jolla, California, United States of America; Massachusetts General Hospital and Harvard Medical School, United States of America

## Abstract

Here we present the first global functional analysis of cellular responses to pore-forming toxins (PFTs). PFTs are uniquely important bacterial virulence factors, comprising the single largest class of bacterial protein toxins and being important for the pathogenesis in humans of many Gram positive and Gram negative bacteria. Their mode of action is deceptively simple, poking holes in the plasma membrane of cells. The scattered studies to date of PFT-host cell interactions indicate a handful of genes are involved in cellular defenses to PFTs. How many genes are involved in cellular defenses against PFTs and how cellular defenses are coordinated are unknown. To address these questions, we performed the first genome-wide RNA interference (RNAi) screen for genes that, when knocked down, result in hypersensitivity to a PFT. This screen identifies 106 genes (∼0.5% of genome) in seven functional groups that protect *Caenorhabditis elegans* from PFT attack. Interactome analyses of these 106 genes suggest that two previously identified mitogen-activated protein kinase (MAPK) pathways, one (p38) studied in detail and the other (JNK) not, form a core PFT defense network. Additional microarray, real-time PCR, and functional studies reveal that the JNK MAPK pathway, but not the p38 MAPK pathway, is a key central regulator of PFT-induced transcriptional and functional responses. We find *C. elegans* activator protein 1 (AP-1; c-jun, c-fos) is a downstream target of the JNK-mediated PFT protection pathway, protects *C. elegans* against both small-pore and large-pore PFTs and protects human cells against a large-pore PFT. This in vivo RNAi genomic study of PFT responses proves that cellular commitment to PFT defenses is enormous, demonstrates the JNK MAPK pathway as a key regulator of transcriptionally-induced PFT defenses, and identifies AP-1 as the first cellular component broadly important for defense against large- and small-pore PFTs.

## Author Summary

The plasma membrane surrounds cells and protects their interior from the environment and from attack by disease-causing agents like bacteria and viruses. Bacteria that cause disease have discovered that an effective way to attack cells is to secrete proteins (pore-forming toxins) that breach, *i.e.*, form holes in, the plasma membrane. How cells deal with and survive this kind of attack is poorly understood. Here, we report on the first large-scale study of the genes and mRNA transcripts that respond to pore-forming toxin attack in cells. We find that a remarkable portion, >0.5%, of the cell's genome protects it against pore-forming toxins. These data led us to look more closely at mitogen-activated protein kinase pathways as regulators of pore-forming toxin defenses. We find that half of the PFT-induced protective response is controlled by a single, conserved signaling pathway in cells, which controls a complex array of downstream targets and which protects against both large pore and small pore toxins. Our results indicate that defense against pore-forming toxins is a very ancient defense that utilizes a much more complex and extensive response in cells than previously demonstrated.

## Introduction

Pore-forming toxins (PFTs) are proteinaceous virulence factors that play a major role in bacterial pathogenesis [1], [2], [3]. PFTs constitute the single largest class of bacterial virulence factors, comprising ∼25–30% of all bacterial protein toxins [3], [4]. PFTs have been demonstrated to be important *in vivo* virulence factors for *Staphylococcus aureus*, Group A and B streptococci, *Streptococcus pneumoniae*, *Enterococcus faecalis*, uropathogenic *Escherichia coli*, *Clostridium septicum*, and *Vibrio cholerae* [2], [5], [6], [7], [8]. There are various ways of grouping PFTs, based on the structure of the pore, based on their pore size as determined by structural or functional data, or even based on the organisms that produce them [9], [10]. With regards to pore size, they generally can be grouped into two categories, those that form small (1–2 nm) diameter pores and those that form large (≥30 nm) diameter pores [9], [10]. Regardless of these groupings, PFTs as a class play a singularly important role in bacterial pathogenesis in mammals.

The mode of PFT action is simple yet elegant—they poke holes in the plasma membrane, breaching cellular integrity and disrupting ion balances and membrane potential [10]. As a consequence, cells die or malfunction, which significantly aids bacterial pathogenesis. Although unregulated pores at the cell surface might be expected to be catastrophic, cells have apparently evolved some mechanisms to protect against low-moderate doses of PFTs. First shown in *Caenorhabditis elegans* and then demonstrated in mammalian cells, the p38 mitogen-activated protein kinase (MAPK) pathway was the first intracellular pathway demonstrated to protect cells against PFTs [11], [12], [13], [14]. *C. elegans* animals or mammalian cells lacking p38 MAPK are more susceptible to killing by PFTs. Three different downstream targets of the p38 PFT defense pathway were identified in *C. elegans*—two toxin-regulated targets of MAPK called *ttm-1* and *ttm-2*, and the IRE-1 – XBP-1 unfolded protein response (UPR) pathway [11], [14]. The *ttm* genes and the UPR are all required for PFT defenses, are all induced by crystal toxin PFT in *C. elegans*, and all require the p38 pathway for their induction. Conserved induction of the UPR in mammalian cells by a PFT that makes similar in size to crystal toxin was demonstrated [14]. A few other scattered studies have identified hypoxia inducible factor (*hif-1*), *wwp-1* (part of the insulin pathway), and sterol regulatory element binding protein (SREBP) as involved in cellular defenses against PFTs [15], [16], [17].

These studies raise the question as to how extensive cellular defenses to PFT attack are. In a broader sense, since PFTs likely act similar to membrane damage that occurs in daily the life of cell [3], [10], these studies raise the question as to how cells deal with unregulated holes at their membranes. How many genes are involved? Are PFT defenses relatively limited or are they extensive? Is there a coordinated pathway for defensive responses or are multiple parallel pathways involved? Little work has been done in this area since it was assumed that unregulated pores at the membrane are catastrophic, likely leading to osmotic lysis. In essence, PFT attack was assumed to be too simple for detailed scientific research.

To address the extent to which cells respond to PFT attack, we report here on the first high-level systematic study of PFT responses in cells. Namely, we perform a *C. elegans* RNAi screen to characterize on a genome-wide scale the genes involved in PFT defenses. Follow up of this data led us to investigate the relative importance of two MAPK pathways in regulating PFT defenses. The combination of these data with other functional and molecular data using both small- and large-pore PFTs in *C. elegans* and mammalian cells reveal important insights into the extent of their genome that cells employ to neutralize proteinaceous membrane pores and how cellular responses to PFTs are regulated.

## Results

### Genome-Wide RNAi Screen for Genes That Protect Against a Small-Pore PFT

**Figure 1:**
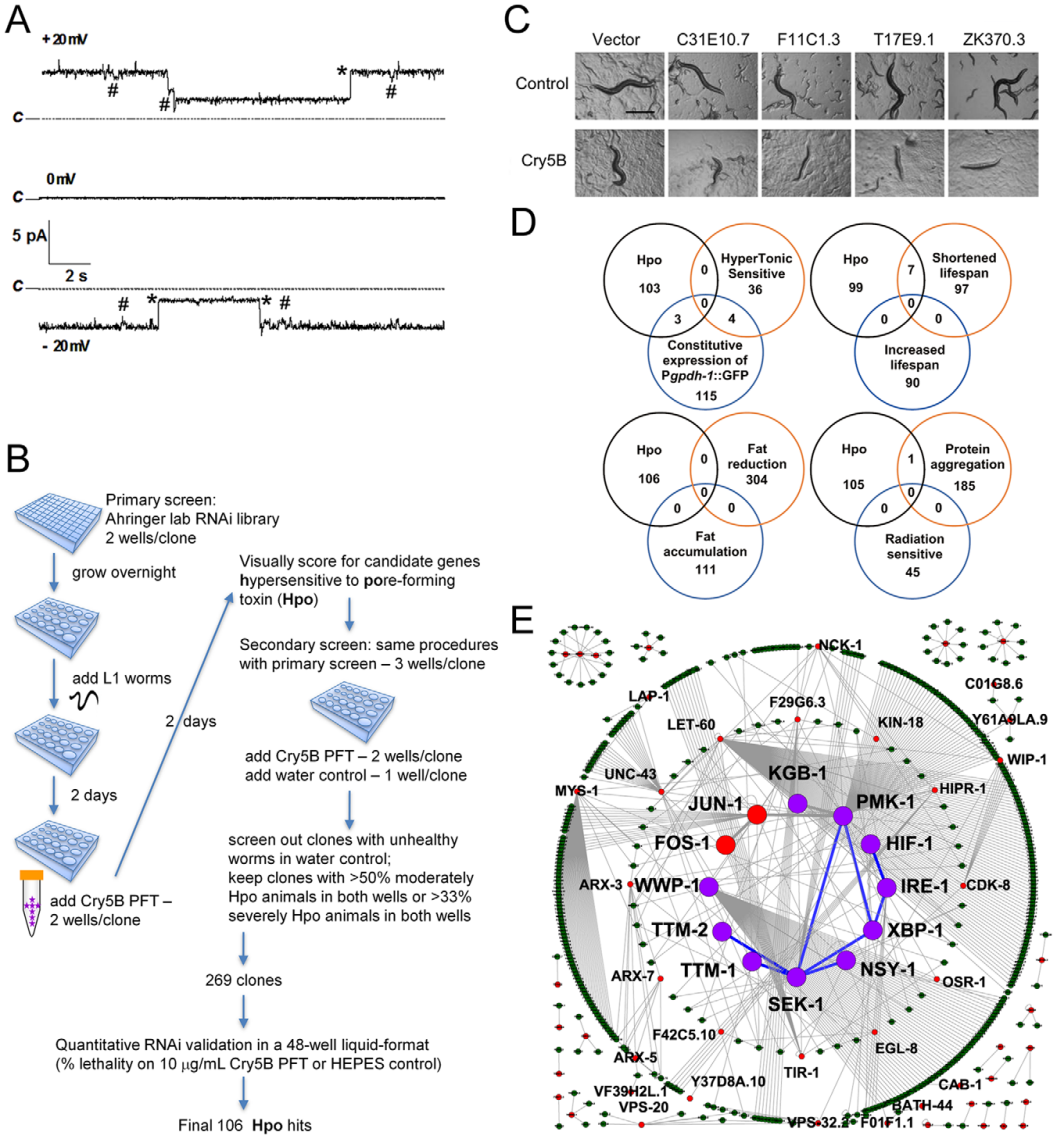
Genome-wide identification of *hpo* genes involved in PFT defenses. (**A**) Cry5B is a PFT. Representative channel current traces recorded at various holding voltages across a planar lipid bilayer into which activated Cry5B (5–10 µg/mL) was inserted under symmetrical 150 mM KCl conditions. Upwards jumps at +20mV and downwards jumps at −20mV correspond to the current flowing in channels in the open state. Dotted lines (marked “C”) indicate the zero current, corresponding to all channels being in their closed state. Records show large jumps (marked with *), corresponding to a channel conductance of 125 pS, and smaller transitions (marked with #) of about 21 pS that may represent subconductance states of the channel. (**B**) Flow chart for genome-wide RNAi screening and validation of genes affecting defense to Cry5B. (**C**) Photographs of typical Hpo hits in the primary screen. Scale bar: 0.5 mm. (**D**) Venn diagrams illustrating the distribution of common genes between the genome-wide Hpo screen and eight other genome-wide RNAi screens in *C. elegans*. (**E**) Putative PFT defense networks assembled from *C. elegans* interactome databases. Purple circles: previously published *hpo* genes. Red circles: *hpo* genes from our RNAi screen. Green circles: other genes connected to *hpo* genes within the network. Blue lines: known interactions for *hpo* genes. Grey lines: interactions based on interactome.

*C. elegans* is susceptible to crystal (Cry) protein PFTs, such as Cry5B, made by the soil bacterium *Bacillus thuringiensis* (Bt) [18], [19]. Based on homology modeling using several available Cry toxin structures as templates [20], Cry5B is a member of the three-domain Bt Cry PFTs that generally form small 1–2 nm diameter pores similar in size to those of α-toxin from *S. aureus* and cytolysin from *V. cholerae* [1], [3], [21]. To directly demonstrate that Cry5B is a PFT, we examined the ability of purified, activated Cry5B to permeabilize artificial phospholipid membranes. Current transitions between open and closed states demonstrate clearly that Cry5B forms ion channels in planar lipid bilayers (Figure 1A), and is therefore a functional PFT. Furthermore, the conductance of the pores is 125pS or less, consistent with that of other Cry toxins tested under identical experimental conditions [22], and whose pore size has been determined to be in the 1–1.3 nm radius [23]. Thus, Cry5B intoxication of *C. elegans* is a valid model of a small-pore PFT attack upon eukaryotic cells *in vivo*.

To ascertain globally how extensive cellular commitment to protection against PFTs is, we performed a genome-wide RNAi screen using the Ahringer bacterial RNAi feeding library, which targets 16000+ *C. elegans* genes or about 85% of the *C. elegans* genome [24] (Figure 1B). Each of the 16000+ individual gene knock-downs were fed Cry5B PFT and assayed in duplicate wells for the Hpo (**h**ypersensitive to **po**re-forming toxin) phenotype (Figure 1C). Hpo animals display a significantly higher level of intoxication compared to wild-type animals when exposed to a sublethal PFT dose. Intoxicated animals are dead or pale, shrunken, and less motile. Several hundred initial hits were retested for the Hpo phenotype in a second round of semi-quantitative knock-down assays (see Materials and Methods). At this round, each gene knock-down was also counter assayed for health in the absence of PFT attack; RNAi clones that caused obvious ill health in the absence of PFT were eliminated from future consideration. RNAi treatments that replicated the Hpo phenotype in the second round were then subjected to a third round of testing, namely quantitative mortality assays in the presence or absence of a fixed dose of Cry5B PFT (10 µg/mL, triplicate wells). We kept genes for which knock down resulted in ≤60% viability on 10 µg/mL PFT relative to empty vector controls and ≥83% viability in the absence of PFT relative to empty vector controls. These criteria were selected to maintain a balance between gene knock-downs with solid Hpo phenotypes and relatively good health in the absence of toxin.

One hundred and six gene knock-downs passed the three rounds of testing (Table 1). Relative to no knock-down controls, 1) for 92/106 genes, there is ≤10% lethality in the absence of toxin, 2) for 91/106 genes there is ≥50% lethality in the presence of toxin, and 3) there is ≥4X more lethality in the presence of toxin than in the absence of toxin for all but two genes. To demonstrate the robustness of the list, we selected 12 of these *hpo* genes that span the range of PFT hypersusceptibility and, in a fourth round of testing, repeated PFT lethality assays with knock-downs in four independent trials. All twelve knock-downs were statistically confirmed to be Hpo relative to no knock-down controls and healthy in the absence of PFT (Table S1).

**Table 1:** RNAi clones that render the animals hypersensitive to Cry5B PFT.

Sequence name		Gene name^a^	Description	Ortholog and/or Homolog^c^	% Survival on Cry5B^d^	% Survival on HEPES^e^	Double Verification^f^	Cry5B-DEG^g^
G-protein-coupled receptor								
C24B9.7		*srg-59*	GPCR		27	93		
C51F7.2		*srg-45*	GPCR		7	102		
F14F8.5		*srw-43*	GPCR		40	92		
F18E3.6		*srw-71*	GPCR		31	95		
Y46H3C.1		*srw-100*	GPCR		41	99		
Ligand and cell surface related								
Y26D4A.2		*hpo-2*	Cysteine rich domain distantly related to the C-type lectin		39	104		
F33D11.9		*hpo-3*	GPI-anchor attachment protein GAA1	H. M. D.	12	100		
T05E11.6		*hpo-4*	GPI-anchor transamidase	H. M. D.	13	95		
T14G10.7		*hpo-5*	GPI-anchor transamidase	H. M. D.	26	102		
T10D4.4		*ins-31*	Insulin-like peptide		40	105		
Y41D4B.16		*hpo-6*	Mucin-3A	H.	27	103		▴
F11C1.3		*scav-4*	Plasma membrane glycoprotein CD36	H. M. D.	37	99		▾
Signal transduction								
K11E8.1		*unc-43*	Calcium/calmodulin-dependent protein kinase (CaMKII)	H. M. D.	57	100		
F39H11.3		*cdk-8*	Cyclin-dependent serine/threonine protein kinase	H. M. D.	40	94		
Y38C9A.2		*cgp-1*	GTP-binding protein	H. M. D.	4	98		
ZK792.6		*let-60*	GTP-binding RAS protooncogene	H. M. D.	60	99		
T07A9.3		*kgb-1* b	JNK (Jun-N-terminal kinase)-like MAPK	H. M. D.	20	93	√	▴
ZK470.5		*nck-1*	NCK adaptor protein 2	H. M. D.	56	96		
B0348.4		*egl-8*	Phospholipase C beta	H. M. D.	23	84		
T17E9.1		*kin-18*	Protein serine/threonine kinase	H. M. D.	40	87	√	▴
F59A6.1		*nsy-1* b	Protein serine/threonine kinase (MAPKKK)	H. M. D.	48	98		
C40A11.5		*hpo-7*	Protein tyrosine phosphatase	H. M.	47	85		
T15B7.2		*hpo-8*	Protein tyrosine phosphatase	H. M. D.	51	106		
F42C5.10		*ttm-4*	Sarcoplasmic reticulum histidine-rich calcium-binding protein	H.	56	98	√	▴
Y59A8B.23		*gck-3*	Ste20-like serine/threonine protein kinase	H. M. D.	49	87		
F13B10.1		*tir-1* b	SAM domains and Toll-IL-1 receptor domain	H. M. D.	48	96		▴
Transcription factor and gene expression								
F29G9.4		*fos-1*	AP-1 transcription factor	H. M. D.	36	90		▴
T24H10.7		*jun-1*	AP-1 transcription factor	H. M. D.	35	98		▴
C05C8.6		*hpo-9*	BTB/POZ domain	H. M. D.	32	101		
ZC518.3		*ccr-4*	CCR4-NOT transcription complex	H. M. D.	22	101	√	
VC5.4		*mys-1*	Histone acetyltransferase	H. M. D.	37	97		▴
F01F1.1		*hpo-10*	Leucine permease transcriptional regulator	H. M. D.	20	102		
C07D10.2		*bath-44*	Meprin-associated Traf homology (MATH) domain	H. M. D.	16	87	√	
C49H3.5		*ntl-4*	MOT2 transcription factor	H. M. D.	35	84		
T13F3.3		*nhr-127*	Nuclear hormone receptor	H. M. D.	43	97		
H37N21.1		*hpo-11*	Nuclear receptor-binding protein	H. M. D.	46	98		
ZK1193.5		*dve-1*	SATB transcripton factor	H. M. D.	28	83		
Transporter								
Y54G9A.3		*kqt-3*	KCNQ-like K+ channel subunits	H. M. D.	34	101	√	
C42C1.10		*hpo-12*	Mitochondrial solute carrier protein	H. M. D.	17	108		
T24H7.5		*tat-4*	P-type ATPase	H. M. D.	42	104	√	▴
W09C2.3		*mca-1*	Plasma membrane Ca2+ ATPase	H. M. D.	36	91		
R186.5		*shw-3*	Voltage-gated K+ channel KCNB/KCNC	H. M. D.	60	92	√	
Vesicular trafficking								
C23H4.1	*cab-1*		AEX-3 interacting	H. M. D.	18	98		▴
C37C3.3	*vps-32.2*		Charged multivesicular body protein	H. M. D.	12	92		
Y65B4A.3	*vps-20*		Charged multivesicular body protein	H. M. D.	22	99		
Y54E10A.2	*cogc-1*		Conserved oligomeric Golgi complex (COGC)	H. M. D.	10	91		
Y105E8B.2	*exoc-8*		Exocyst complex	H. M. D.	46	96		
Y106G6H.7	*sec-8*		Exocyst complex	H. M. D.	28	105		
T07A5.2	*unc-50*		Golgi component Gmh1p	H. M. D.	29	96		
ZK370.3	*hipr-1*		Huntingtin-interacting protein	H. M. D.	29	94	√	▴
VF39H2L.1	*syx-17*		SNARE protein	H. M. D.	19	105		▴
Metabolism								
Y105E8A.10	*hpo-13*		Bile acid beta-glucosidase	H. M. D.	27	106		
F59G1.1	*cgt-3*		Ceramide glucosyltransferase	H. M. D.	35	92		
C31E10.7	*hpo-14*		Cytochrome B5	H. M. D.	20	96		▾
F28D1.11	*dpm-3*		Dolichol phosphate mannosyltransferase	H.	22	102		
Y54E5A.1	*ttm-5*		Fatty acid desaturase	H. M. D.	45	94	√	▴
C24G6.6	*hpo-15*		Flavin-containing amine oxidase	H. M. D.	51	101		▾
R01H2.5	*ger-1*		GDP-L-fucose synthetase	H. M. D.	33	84		
M02B7.4	*hpo-16*		Glycosyltransferase	H. M. D.	12	99		
R10D12.12	*hpo-17*		Glycosyltransferase	H. M. D.	58	98		
F41E6.14	*oac-29*		Integral membrane O-acyltransferase		25	90		
F32D1.2	*hpo-18*		Mitochondrial F1F0-ATP synthase subunit epsilon	D.	31	104		▾
F20H11.3	*mdh-1*		Mitochondrial malate dehydrogenase	H. M. D.	39	102		▾
ZK353.6	*lap-1*		Mitochondrial zinc metalloprotease leucine aminopeptidase	H. M. D.	48	93		
T05H4.5	*hpo-19*		NADH-cytochrome B5 reductase	H. M. D.	53	100		▾
Y47D3A.17	*obr-1*		Oxysterol-binding protein	H. M. D.	6	96	√	
C10G11.5	*pnk-1*		Pantothenate kinase	H. M. D.	38	86		
ZK809.7	*prx-2*		PeroxiIsomal membrane protein 3	H. M. D.	20	101		
F54F2.8	*prx-19*		Peroxisomal farnesylated protein	H. M. D.	54	96		▾
F39G3.7	*prx-6*		Peroxisome assembly factor 2	H. M. D.	21	100		
Y110A2AL.12	*hpo-20*		Phosphatidylinositol-glycan biosynthesis class W protein	H. M. D.	59	99		
Y37D8A.10	*hpo-21*		Signal peptidase complex	H. M. D.	28	96		
C34C6.5	*sphk-1*		Sphingosine kinase	H. M. D.	11	99		
Miscellaneous								
M01B12.3		*arx-7*	Actin-related protein (Arp)2/3 complex	H. M. D.	30	100		
Y37D8A.1		*arx-5*	Actin-related protein (Arp)2/3 complex	H. M. D.	25	91		
Y79H2A.6		*arx-3*	Actin-related protein (Arp)2/3 complex	H. M. D.	16	85		
B0024.2		*col-150*	Collagen	H. M. D.	37	100		
C08B6.7		*hpo-22*	Dystrophia myotonica-containing WD repeat motif protein	H. M. D.	49	108		
Y48G8AL.1		*hpo-23*	E6-AP ubiquitin-protein ligase	H. M. D.	38	99		
T23D8.7		*hpo-24*	Eukaryotic translation initiation factor 2C 4	H. M. D.	45	87		
F16G10.4		*hpo-25*	Extracellular protein with cysteine rich structures		18	83		
D2024.6		*cap-1*	F-actin capping protein alpha subunit	H. M. D.	42	91		
R09B5.1		*fbxa-195*	F-box protein		52	102		
F46E10.11		*hpo-26*	Fibrillins	H.	39	100		
C34G6.1		*hpo-27*	HEAT repeat	H. M. D.	21	90		
B0336.11		*hpo-28*	Motile sperm domain	H. M. D.	36	93		
Y50D7A.4		*hpo-29*	N-terminal acetyltransferase	H. M. D.	49	100		
C32E12.3		*osr-1*	Osmotic stress response protein		58	97		
F37C12.2		*eip-24*	p53-mediated apoptosis protein EI24/PIG8	H. M. D.	28	97		▴
R04F11.1		*hpo-30*	Tight junction claudin		40	93		
F55B12.4		*hpo-31*	tRNA nucleotidyltransferase/poly(A) polymerase	H. M. D.	43	98		
R144.4		*wip-1*	WAS/WASL-interacting protein family member 3	H. M. D.	7	90		▴
Uncharacterized								
C01G8.6		*hpo-32*	Unknown	H.	46	92		
F28B1.1		*hpo-33*	Unknown		2	86		
F29G6.3		*hpo-34*	Unknown		42	97		▾
F45H11.3		*hpo-35*	Unknown		26	94		▾
T27C4.2		*hpo-36*	Unknown		48	105		
Y47D7A.10		*hpo-37*	Unknown		56	104		
Y50D7A.5		*hpo-38*	Unknown		25	95		
Y54E10A.16		*mab-31*	Unknown		51	98		
Y61A9LA.9		*hpo-39*	Unknown		46	94		
ZK856.12		*hpo-40*	Unknown		48	98		
C40H5.6		*hpo-41*	Pseudogene		46	93		
F13A7.6		*sri-64*	Pseudogene		50	101		
T10C6.8		*hpo-42*	Pseudogene		31	95	√	
Y18D10A.18		*fbxa-121*	Pseudogene		40	104		

a The gene names were annotated based on WormBase release WS221. All the previously unnamed genes were named either *ttm* or *hpo* based on their phenotypes.

b We have published ten genes that upon reduction lead to a Hpo phenotype and knew of one other gene in the p38 pathway (*tir-1*) that is a strong *hpo*. Of these 11, three are not in the Ahringer RNAi library (*wwp-1*, *sek-1*, *pmk-1*); one (*ire-1*), upon RNAi, leads to sickly worms (would have been screened out). Of the remaining seven genes, four have relatively weak to moderate Hpo phenotypes upon knock down (*ttm-1*, *ttm-2*, *hif-1*, and *xbp-1*) were not found (likely missed due to the lack of sensitivity associated with a genome-wide RNAi experiment). All three of the remaining genes (*nsy-1*, *tir-1*, *kgb-1*) that upon knock down result in a strong Hpo phenotype and are otherwise healthy were identified.

c H., M., and D. refer to *Homo sapiens*, *Mus musculus* and *Drosophila melanogaster*, respectively.

**d, e:** % survivals are relative to empty vector controls.

f √ indicates the RNAi clones further confirmed in another round of quadruplicate liquid RNAi assays as shown in Table S1.

g “▴” indicates induction by Cry5B and “▾” indicates repression by Cry5B in 3-hour microarray with a 1.5 fold cutoff. DEG = differentially expressed genes.

Interestingly, of the eight known *hpo* genes identified in previous screens and present in the RNAi library, we found all three of the genes that mutate to a strong Hpo phenotype and none of the four genes that mutate to a weak to moderate Hpo phenotype (see Table 1 legend; knock down of the eighth gene is sickly in the absence of toxin and would have been screened out). Thus, we have likely identified many of the genes that mutate to a strong Hpo phenotype and likely missed an equally large number of genes that mutate to a weak-moderate Hpo phenotype.

The 106 strong *hpo* genes constitute >0.5% of the *C. elegans* genome (∼20,000 genes). Based on the Wormbase annotation, we manually classified them into the following nine categories: G-protein-coupled receptor (GPCR), ligand and cell surface related, signal transduction, transcription factor and gene expression, transporter, vesicular trafficking, metabolism, miscellaneous, and uncharacterized (Figure S1). Thirteen of these genes have been previously implicated in, or are homologues of genes involved in innate immunity, indicating that much of the defense against PFTs is uncharacterized relative to established immune pathways. The most highly represented group is that containing genes associated with metabolic activities, suggesting cells may alter metabolism to mount an appropriate defense and repair in response to PFTs. Among the 106 *hpo* genes, we also identified many genes associated with vesicle trafficking and membrane transporters, both of which have been previously implicated, but not demonstrated to be involved, in PFT defenses [25], [26]. Other significant groupings include GPCR, signal transduction, and transcription factor and gene expression. Such genes might include key components for the sensing of upstream signals, and downstream effector genes for PFT defenses [11], [13]. We found that the majority of these 106 genes have orthologs or homologs in fly (71%), mouse (71%), and humans (75%) (Table 1). Taken together, these findings support the idea that metazoans employ an extensive and conserved response to counteract PFTs and breaches of the plasma membrane.

To understand how functional responses against PFTs compare to responses to other stressors and conditions, we compared our list of 106 *hpo* genes with published genome-wide RNAi screens in *C. elegans* for hypertonic stress sensitivity [27], osmo-regulation [28], life-span regulation [29], [30], fat content regulation [31], protein aggregation regulation [32] and irradiation sensitivity [33] (no other screens involving a challenge related to bacterial pathogenesis have been published to our knowledge) (Figure 1D). The comparison revealed a statistically significant (*P* = 0.000051) overlap of the *hpo* genes with only one set of genes, those that upon inactivation lead to reduced life-span [30]. The lack of overlap with most other biological processes suggests that protection against small pores involves a specialized protective response and/or integrates many different responses, none of which are singularly dominant.

### c-Jun N-Terminal Kinase (JNK)-like MAPK but not p38 MAPK is a Master Regulator of Cry5B PFT-Induced Transcriptional Responses

To make sense of these 106 genes, we mapped potential interactions among them (along with a few others from our previous publications) using the *C. elegans* interactome database that contains protein-protein interactions and genetic interactions based on yeast-2-hybrid data and literature [34]. Using this unbiased approach, a major interconnected network containing one-third (38) of all the *hpo* genes emerges. This network contains at its center two MAPK pathways, the p38 MAPK pathway and the c-Jun N-terminal kinase (JNK) MAPK pathway (Figure 1E). Thus, based on interactions from our genome-wide RNAi screen, we hypothesized that MAPK pathways play a central role in cellular PFT responses.

The identification of p38 MAPK as important in PFT defenses in *C. elegans*, insect, and mammalian cells has been noted above and characterized in detail [11], [12], [13], [14]. The conservation of the p38 pathway in PFT defenses suggested it might be the central regulator of these defenses. On the other hand, the JNK-like MAPK pathway, in *C. elegans* represented by the JNK-like MAPK KGB-1, was briefly noted as playing a role in Cry5B defenses [11] but not functionally studied in detail in any system. Whether it might be central to PFT defenses or a peripheral player was unknown. The quantitative data from our genome-wide RNAi screen suggested *kgb-1* might be important since knock-down results in a quantitatively strong hypersensitivity to PFT phenotype (Table 1, Table S1). Knock-down in the MAPKK gene, *mek-1*, known to function upstream of KGB-1 JNK-like MAPK [35], also results in hypersensitivity to PFT (Figure S2).

To determine the relative importance of the JNK and p38 pathways in PFT defenses, we turned to microarray analyses. Since inductive transcriptional responses are an important part of MAPK-mediated responses, we hypothesized that, if important for PFT defenses, KGB-1 might play an important role in PFT-induced gene transcription.

We previously used microarray analyses to determine to what extent the p38 MAPK pathway controlled Cry5B PFT-induced transcriptional responses and to identify two downstream targets of the p38 pathway, *ttm-1* and *ttm-2*, both of which phenotypically play a minor role in Cry5B PFT defenses [11]. Here, we carried out a similar microarray study with the JNK MAPK pathway in order to determine how many gene transcripts that are normally induced by Cry5B PFT are dependent upon the JNK pathway for their induction. We exposed wild-type and *kgb-1(um3)* JNK-like MAPK mutant animals either to Cry5B PFT-expressing bacteria or to bacteria carrying the empty vector (no-toxin control), each for three hours. The *kgb-1(um3)* loss-of-function mutant lacks 1.2 kb of genomic DNA and deletes most of the kinase domain [36], [37]. That *kgb-1(um3)* represents a null mutant is supported by our Western blotting experiments (see below). RNA was isolated from these animals in three independent repeats and hybridized to Affymetrix-based *C. elegans* whole genome microarrays. We then compared these data with those found in the p38 MAPK microarray experiment [11].

**Figure 2:**
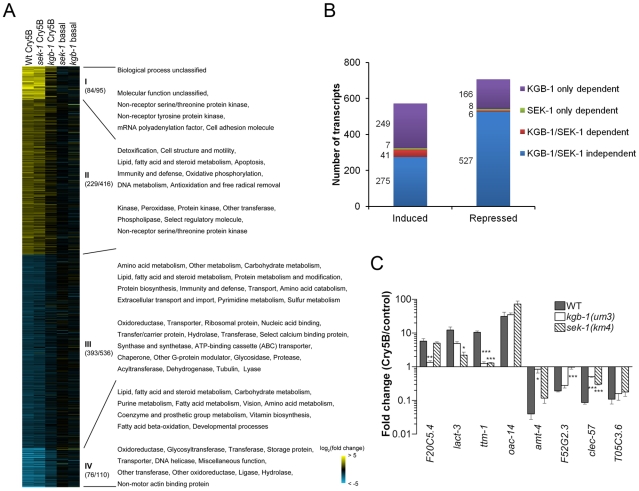
MAPK pathway-dependent transcriptional responses to PFT attack. (**A**) Heat-map of PFT-responsive genes in microarray experiments. Cry5B responsive genes with ≥2-fold induction (yellow) or repression (blue) relative to non-treated animals are shown. Brighter shades of color correspond to greater fold changes in expression. Different comparisons of treatments are presented at the top of each column. Wt Cry5B: Fold change in Cry5B-treated/non Cry5B-treated *glp-4(bn2)* animals. sek-1 Cry5B: Fold change in Cry5B-treated/non Cry5B-treated *glp-4(bn2);sek-1(km4)* animals. kgb-1 Cry5B: Fold change in Cry5B-treated/non Cry5B-treated *glp-4(bn2);kgb-1(um3)* animals. sek-1 basal: Fold change in *glp-4(bn2);sek-1(km4)*/*glp-4(bn2)* animals (both non Cry5B-treated). kgb-1 basal: Fold change in *glp-4(bn2);kgb-1(um3)*/*glp-4(bn2)* animals (both non Cry5B-treated). *glp-4(bn2)* animals lack a functional germline and have otherwise normal response to Cry5B (see Materials and Methods). Use of these animals removes a major tissue from the animals, allowing for intestinal mRNAs to represent a larger portion of the total RNA population. The values in the parenthesis after the slash indicating the number of the genes in each cluster (I and IV strongly up- or down-regulated; II and III moderately up- or down-regulated), with the values before slash denoting the genes with known PANTHER ontologies. The corresponding enriched PANTHER biological processes and molecular functions are shown in each cluster indicated with Roman numerals. For genes involved in immunity/defense processes, we found 14 in cluster II and 22 in cluster III. (**B**) Summary of number of genes up- and down-regulated by Cry5B, and their dependence upon KGB-1 and SEK-1. (**C**) qRT-PCR analysis of selected genes from microarray analysis. Results are the average of three experiments and error bars are standard error of the mean. Each gene behaved in the qRT-PCR experiment as was found in the microarray experiment. In this and other figures, * for *P*≤0.05, ** for *P*≤0.01 and *** for *P*≤0.001.

From the total of six wild-type expression profiling microarrays (three repeats for the *sek-1* set and three for the *kgb-1* set, both with and without toxin), we found that at a 2-fold cut-off, 572 transcripts were induced in wild-type *C. elegans* upon exposure to Cry5B PFT; that number increases to 1117 transcripts if the cut-off is set to 1.5-fold (*P*<0.01 for both). Of repressed transcripts, we found that 707 transcripts repressed at the 2-fold cut-off and 1428 repressed at the 1.5-fold cut-off (*P*<0.01 for both) (Figure 2A; see Supporting Information S1 for a complete list of differentially expressed transcripts). We cross-checked these transcripts with the 106 *hpo* genes identified above, and found that 15 *hpo* genes were induced ≥1.5 fold and 8 were induced ≥2 fold. Statistically, there is a significant enrichment of induced genes on the *hpo* list (*P* = 0.00041 and *P* = 0.0148 respectively). In contrast, the overlap between repressed genes and *hpo* genes is not significant (*P* = 0.345 with 1.5 fold cutoff, seven gene overlap, and *P* = 0.652 with 2 fold cut-off, four gene overlap), indicating that the appearance of repressed genes on the list of *hpo* genes is likely from random chance.

**Figure 3:**
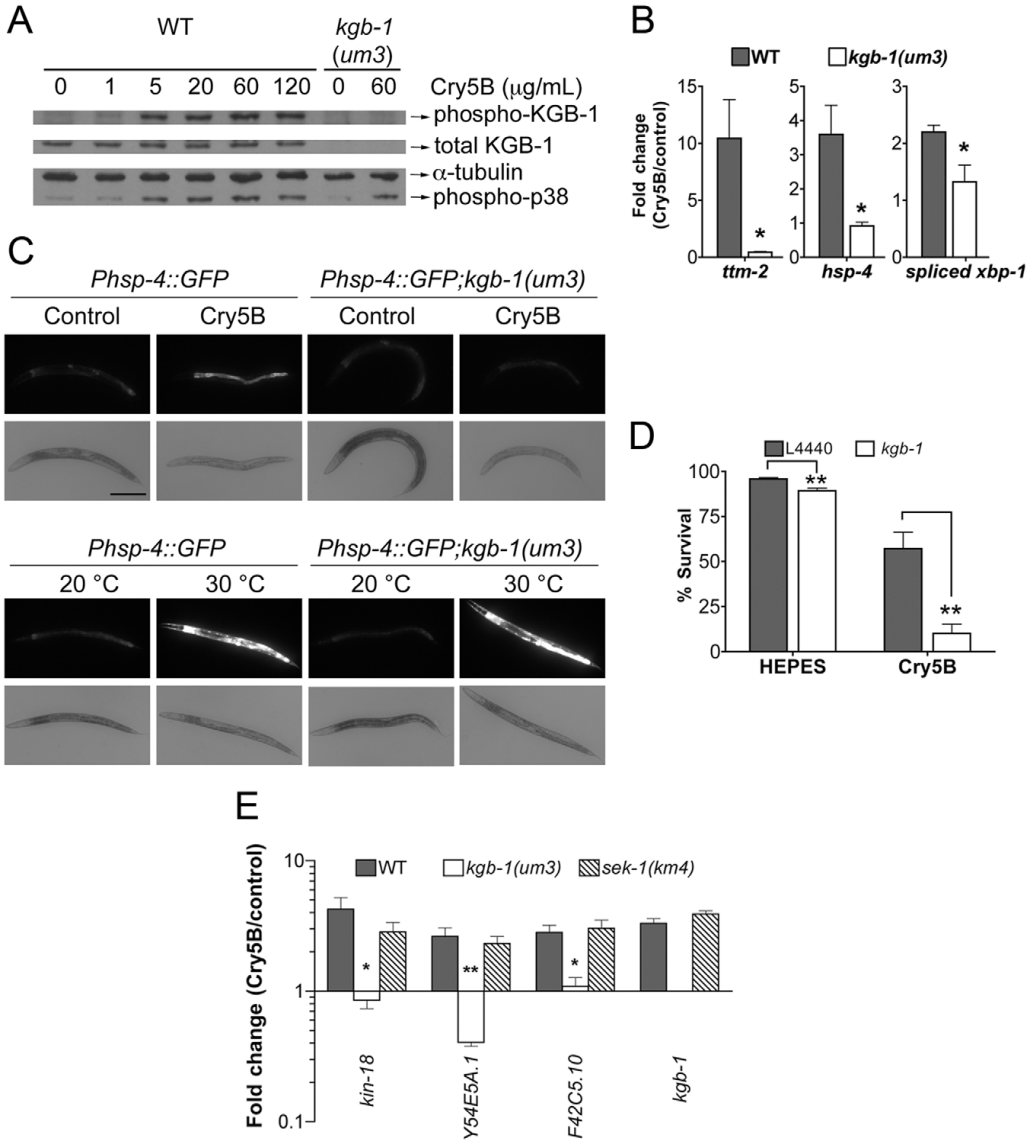
KGB-1 JNK-like MAPK regulates p38-dependent and -independent pathways involved in PFT defenses. (**A**) Activation of KGB-1 by Cry5B PFT. Activation was assessed by Western blotting using antibodies specific for phosphorylated and total KGB-1. α-tubulin served as an equal-loading control and phosphorylated p38 MAPK was also tested. Specificity of the antibody was verified by probing *kgb-1(um3)* mutant animals. The experiment shown is representative of three experiments with similar results. (**B**) qRT-PCR analysis of known p38 MAPK downstream target genes in response to PFT. All three target genes are regulated by KGB-1. Average of three experiments, error bars are standard error of the mean. (**C**) *In vivo* induction of P*hsp-4*::GFP in the intestine by Cry5B requires KGB-1. The strains *Phsp-4*::GFP and *Phsp-4*::GFP;*kgb-1(um3)* were fed either control *E. coli* or *E. coli* expressing Cry5B for 8 hours or shifted to 30°C for 5 hours in the heat shock treatment. Cry5B induces GFP within the intestinal cells of the strain *Phsp-4*::GFP but not in the strain containing the *kgb-1(um3)* mutant. General stress induction of *Phsp-4*::GFP via heat shock is independent of KGB-1. The experiment was performed three times and representative worms are shown. Scale bar is 0.2 mm. (**D**) Intestinal *kgb-1* is required for protection against Cry5B PFT. Intestine-specific knockdown of *kgb-1* in VP303 animals are quantitatively hypersensitive to purified Cry5B, compared to empty vector control (L4440). Results are the average of three different experiments; error bars indicate standard error of the mean. (**E**) qRT-PCR analysis of four KGB-1-dependent *hpo* genes in response to PFT. All these genes show a KGB-1 dependent/SEK-1 independent induction by Cry5B PFT. Average of three experiments, error bars are standard error of the mean.

We then classified the dependence of all the transcripts (≥2-fold cut-off) into one of four categories: 1) transcripts dependent upon only the p38 MAPK pathway for their induction or repression by Cry5B PFT, 2) transcripts dependent upon only the JNK MAPK pathway for their induction or repression by Cry5B PFT, 3) transcripts dependent upon both p38 and JNK MAPK pathways for their induction or repression, and 4) transcripts whose induction or repression by Cry5B PFT are independent of either MAPK pathway (Figure 2B). The results of these analyses were stunning: whereas the p38 MAPK pathway was responsible for regulating 8% (48/572) and 2% (14/707) of the PFT-induced and -repressed transcripts respectively, the JNK MAPK pathway controlled 51% (290/572) and 24% (172/707) of the PFT-induced and -repressed transcripts respectively. Thus, the JNK pathway controls more than half of the induced transcripts and more than six times the total number of transcripts controlled by the p38 pathway. Furthermore, the JNK pathway controls 85% (41/48) of the p38 MAPK-dependent induced transcripts and 43% (6/14) of these p38-dependent repressed transcripts. To validate the microarray data, we chose one up- and one down-regulated gene from each of the four categories, isolated RNA from three independent experiments (minus or plus Cry5B PFT), and performed quantitative real-time (qRT) PCR analyses (Figure 2C). The qRT-PCR results validate the microarray results. Since the JNK MAPK pathway is important for the PFT-induced transcripts, we predicted the pathway itself would be activated upon exposure to PFT. We first showed that there is no detectable KGB-1 protein in *kgb-1(um3)* mutant animals and further confirmed that the JNK-like MAPK pathway is activated in *C. elegans* treated with Cry5B PFT (Figure 3A), as has been seen with the mammalian cell responses to PFTs [38], [39]. Interestingly, in *kgb-1(um3)* mutant animals, we detected the phosphorylated form of p38 MAPK (Figure 3A) induced by Cry5B PFT indicating that the p38 MAPK activation is not directly regulated by KGB-1.

In addition to the fact that JNK MAPK is important for induction of most of the PFT-induced transcripts, including those regulated by p38, we wanted to know if JNK MAPK is also important for induction of PFT-induced transcripts that are functionally important (*e.g.*, knock-down to Hpo phenotype). We therefore tested whether or not KGB-1 regulates p38-controlled transcripts/pathway that play a role in PFT defenses, namely *ttm-1* and *ttm-2* and the UPR [11], [14]. We find that the induction of both *ttm-1* (Figure 2C) and *ttm-2* (Figure 3B) in response to PFT is dependent upon the JNK MAPK pathway. Cry5B PFT-induced activation of the UPR PFT defense pathway is also dependent upon JNK MAPK, as ascertained by induction of the downstream transcriptional target *hsp-4* transcript levels and by induction of *xbp-1* splicing (Figure 3B). As with p38 MAPK-dependent activation of the UPR by PFT [14], JNK-dependent UPR activation occurs in the intestine in response to Cry5B PFT (the tissue directly targeted by the PFT; Figure 3C, upper panels) but not in response to an unrelated stressor like heat (Figure 3C, lower panels). The UPR-activation data suggest that the JNK pathway functions cell autonomously in the intestine for PFT defenses. To confirm this, we performed intestine-specific RNAi of *kgb-1*, and found that animals lacking KGB-1 in the intestine are hypersensitive to Cry5B PFT (Figure 3D). Thus, the JNK MAPK pathway regulates all currently known p38-dependent PFT protection genes in the target tissue of the PFT.

We next set out to determine, apart from known p38-dependent PFT defense genes, what other defense genes JNK MAPK might regulate. For this information, we turned back to our genome-wide RNAi list of *hpo* genes, examined all eight of the genes that are induced by PFT (2X cut-off), and asked, based on our microarray data, if their induction is dependent upon either the p38 or JNK MAPK pathways. We found four of eight (50%) induced *hpo* genes (namely *kin-18*, *Y54E5A.1*, *F42C5.10* and *kgb-1* itself) require JNK MAPK for their induction whereas none of the eight genes require the p38 MAPK pathway for their induction. The dependence of these four genes upon the KGB-1 and not the p38 MAPK pathway for their induction was confirmed via qRT-PCR (Figure 3E). The same conclusion is reached if one examines genes required for PFT defenses induced at the 1.5-fold cutoff: 7/15 or ∼50% of these genes are dependent upon JNK; none are dependent upon SEK-1. Although the p38 MAPK pathway has been studied in more detail, these data taken together indicate that the JNK MAPK pathway plays a more central role in coordinating induced PFT defenses.

Since KGB-1 is a master regulator of ∼50% of Cry5B PFT-responsive genes, including those that are functionally relevant, we were interested to see how susceptible *kgb-1* mutant animals are to Cry5B PFT attack. Using a dose-dependent lethality assay, we determined the LC_50_ (lethal concentrations at which 50% of the animals die) of the wild-type, *sek-1(km4)* p38 MAPKK-minus, and *kgb-1(um3)* JNK-like MAPK-minus animals treated with Cry5B PFT for 8 days. We find that both *kgb-1(um3)* and *sek-1(km4)* mutant animals have similar LC_50_ values and are both significantly hypersensitive to PFT relative to wild type animals (Table 2). These results were unexpected since, based on the fact that KGB-1 regulates more PFT-induced genes functionally required for PFT protection than the p38 pathway, we predicted that *kgb-1* mutant animals would be more hypersensitive to PFT than *sek-1* mutant animals. There are several explanations for this finding. First, it might suggest that KGB-1, but not SEK-1, transcriptionally regulates many genes required for induced PFT defenses and also some genes that inhibit PFT defenses. Such genes could be temporally segregated—*e.g.*, KGB-1 JNK-like MAPK could initially up-regulate transcriptional responses that are protective (our microarray data is taken at 3 hr post-PFT treatment) and later down-regulate responses that inhibit protection (our LC_50_ data is taken at 8 days). Eventual down-regulation of protection is an important part of induced immune responses and serves to protect cells from the adverse effects of a prolonged or overwhelming immune response. Such positive and negative regulation of a cellular response by the JNK pathway is with precedent [40], [41]. Second, it might also suggest that whereas KGB-1 JNK-like MAPK is a master regulator of transcriptionally-induced PFT defenses, the p38 pathway may play an important role in other (*e.g.*, constitutive) PFT defenses, hence resulting in a more severe *sek-1* phenotype than predicted.

**Table 2:** LC_50_ values along with 95% confidence intervals.

Strain	LC_50_ (µg/mL)	95% CI of LC_50_	Relative Sensitivity LC_50_
WT	10.40	9.54–11.34	
*kgb-1(um3)*	0.084	0.069–0.101	123.8
*sek-1(km4)*	0.070	0.065–0.076	148.6

Relative sensitivity was calculated by LC_50_ wild type/LC_50_ mutant.

### JNK MAPK Protects Against Large-Pore PFTs

Given its importance in the hierarchy of coordinating transcriptionally-induced defenses against a small-pore PFT and its activation in both *C. elegans* treated with small-pore PFT (our data; see above) and mammalian cells treated with large-pore PFT (referenced above), we hypothesized that JNK MAPK might broadly coordinate defenses against PFTs—*i.e.*, it might be required for defense against both small- and large-pore PFTs. To date, no gene has been shown to play a protective role against both large- and small-pore PFTs.

We therefore tested whether a large-pore PFT could intoxicate *C. elegans* using streptolysin O (SLO). SLO binds to cholesterol in cell membranes, forms large 30 nm diameter pores (versus 1–2 nm pores for Cry PFTs), and is an important virulence factor of human-pathogenic streptococci [42]. The repair of the pores formed by SLO in mammalian cells membranes is calcium dependent [25], and a lytic-mutant N402 SLO has been shown to be defective in generating pores in membranes [43]. Whether SLO or any other cholesterol-dependent cytolysin affects *C. elegans* has not been previously reported.

**Figure 4:**
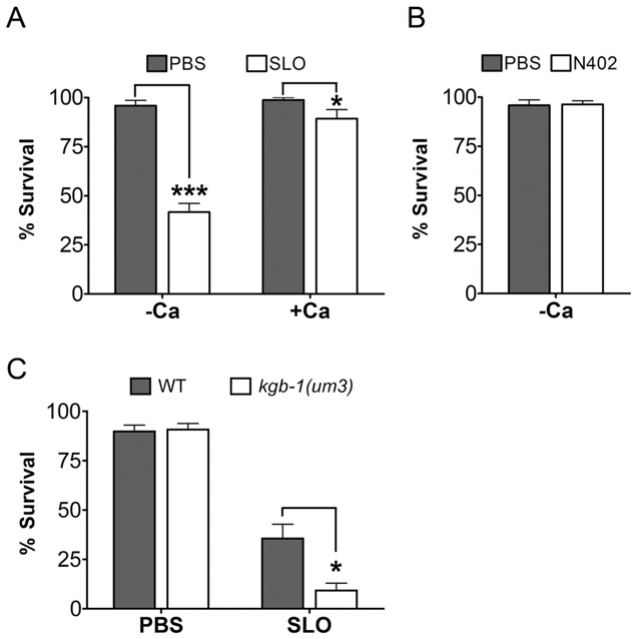
Intoxication of *C. elegans* by SLO and the requirement of *kgb-1* in protecting *C. elegans* against SLO. (**A**) Viability of wild-type animals is significantly lower than wild-type animals on the mammalian PFT SLO in calcium-free S-medium. In calcium-complete medium, SLO shows a dampened toxicity to wild-type animals. (**B**) The mammalian PFT SLO mutant, N402, does not intoxicate wile-type *C. elegans* in calcium-free S-medium. (**C**) JNK-like MAPK protects against mammalian PFTs. Viability of *kgb-1 (um3)* loss-of-function animals is significantly lower than wild-type animals on the mammalian PFT SLO in calcium-free S-medium. Results are the average of three different experiments; error bars indicate standard error of the mean.

We find that wild-type *C. elegans* are intoxicated by wild-type SLO at 50 µg/mL with 58% of animals killed in calcium-free medium and 11% killed in calcium-containing medium in 6 days (Figure 4A). The inhibitory effect of calcium in SLO-mediated killing of *C. elegans* is consistent with known effects of SLO on mammalian cells [25]. Furthermore, the killing of *C. elegans* by SLO is completely dependent upon pore formation since wild-type worms are not killed by the non-lytic SLO mutant N402 (Figure 4B). Thus, the interaction of SLO with *C. elegans* parallels that of the toxin with mammalian cells and the intoxication of *C. elegans* by SLO is a valid model of large-pore PFT intoxication.

We then tested whether or not JNK is required for defense against SLO in *C. elegans*. Wild-type animals and *kgb-1(um3)* mutant animals were exposed to SLO at a dose of 50 µg/mL. Significantly more *kgb-1(um3)* mutant animals are killed by SLO than wild-type animals (Figure 4C), demonstrating that the JNK MAPK pathway is also required for defense against a large-pore PFT.

### AP-1 is a Key Downstream Target of the JNK MAPK PFT Protection Pathway Against Both Small- and Large-Pore PFTs and in Mammalian Cells

*C. elegans fos-1* and *jun-1* were found in our genome-wide RNAi screen as genes important for defense against Cry5B PFT (Table 1). Together, these proteins make up the heterodimeric transcription factor known as activating protein 1 (AP-1). AP-1 is a well-defined regulator of innate immunity and stress responses in mammalian cells and a known downstream target of JNK MAKP pathway in these responses [44]. To date, neither *jun-1* nor *fos-1* has been demonstrated to be involved in protective responses in *C. elegans* nor in PFT protection in any system.

**Figure 5:**
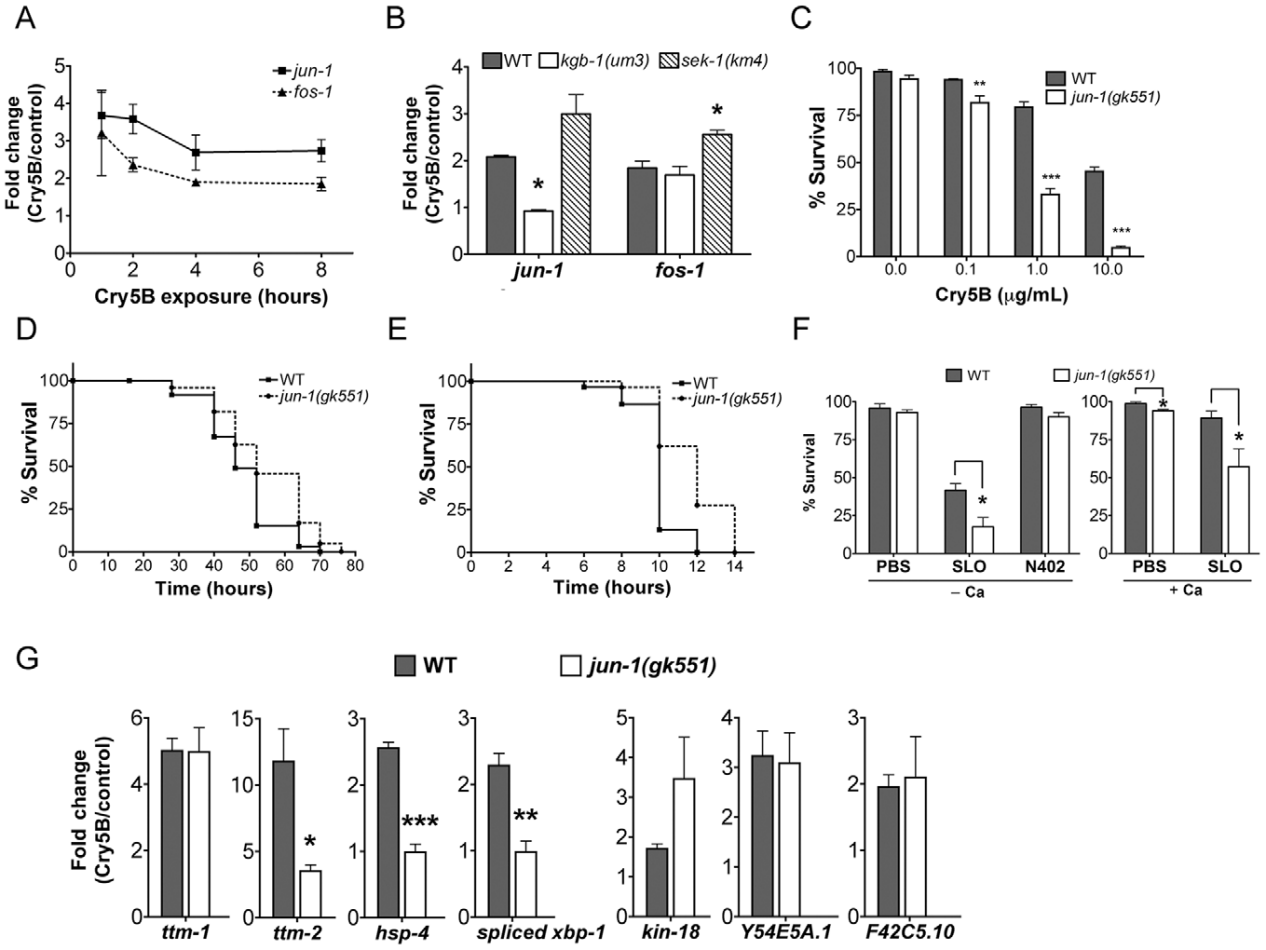
Induction of jun and fos transcripts by Cry5B PFT and specificity of *jun-1* in protecting *C. elegans* against PFTs. (**A**) qRT-PCR results showing time-course of induction of *jun-1* and *fos-1* by Cry5B PFT. (**B**) Cry5B-induced *jun-1* expression is KGB-1 dependent and SEK-1 independent. Data shown were based on 3 hours of PFT treatment. For A and B, three RNA samples independent from each other and from the microarray experiments. (**C**) Viability of wild-type and *jun-1(gk551)* animals on three different doses of Cry5B PFT. (**D, E**) Viability of wild-type and *jun-1(gk551)* animals on (**D**) heat stress and (**E**) *P. aeruginosa* PA14. For A, B, and C results are the average of at least three different experiments; error bars indicate standard error of the mean. For D and E, the data presented are a single representative from three independent assays. (**F**) Viability of wild-type and *jun-1(gk551)* loss-of-function animals on the mammalian PFT SLO and N402 SLO in calcium-free S-medium and calcium-containing S-medium. Results are the average of at least three different experiments; error bars indicate standard error of the mean. (**G**) Three known p38 MAPK downstream target genes in response to Cry5B-PFT are regulated via JUN-1. Cry5B-induced *ttm-2*, *hsp-4* and spliced *xbp-1* expression is JUN-1 dependent. Cry5B-induced *ttm-1*, *kin-18*, *Y54E5A.1* and *F42C5.10* was not attenuated in *jun-1* mutant animals. Results are the average of three experiments, and error bars are standard error of the mean.

Both *C. elegans* AP-1 homologs, *fos-1* and *jun-1* were found to be up-regulated by Cry5B PFT in our microarray study (*P*<0.001). To confirm their induction, we performed qRT-PCR analysis of *C. elegans jun-1* and *fos-1* expression upon treatment with PFT at 1, 2, 4, and 8 hr. Both are robustly induced by Cry5B, with maximum induction at the earliest time point tested (Figure 5A). *jun-1* induction by Cry5B PFT is KGB-1-dependent and SEK-1 independent whereas *fos-1* induction is independent of both (Figure 5B). These inductions are consistent with our microarray data (1.5-fold cut-off) and demonstrate that *jun-1* is a downstream target of KGB-1 during PFT protective responses.

To quantitate the protective function of AP-1 during small-pore PFT attack, we performed Cry5B PFT mortality assays using the available *jun-1* mutant, *jun-1(gk551)* (as opposed to *jun-1* RNAi; the *jun-1(gk551)* allele is likely a null as it deletes 1.4 kb of DNA from the *jun-1* locus, including the DNA binding domain). We found that *jun-1(gk551*) animals are significantly more sensitive than wild-type animals at all doses of Cry5B tested and overall are ∼10 fold more sensitive to PFT (Figure 5C). This level of *jun-1(gk551)* hypersusceptibility to PFTs is greater than that of mutants in other non-MAPK *hpo* genes quantitated to date (namely, *xbp-1*, *hif-1*, *ttm-1*, *ttm-2*, *wwp-1*). We also exposed *jun-1(gk551)* mutant animals to heat stress, and find that, compared to wild-type animals, they are actually slightly resistant to heat stress (Figure 5D; Table S2; Figure S3). When tested against a second form of pathogenic attack, namely *Pseudomonas aeruginosa* PA14, we find that *jun-1(gk551)* mutant animals are resistant relative to wild-type animals (Figure 5E, Table S2, Figure S4). Thus, the sensitivity of *jun-1* mutant animals to PFT is not due to generally compromised health and there is specificity in its role as protecting against PFTs.

To determine whether *C. elegans jun*, like JNK MAPK, is protective against large-pore PFTs, we treated *jun-1(gk551)* animals with 50 µg/mL SLO and compared the percentage killed to wild-type animals exposed to the same dose. Animals lacking *jun-1* are hypersensitive to SLO PFT in the absence or presence of calcium, with 82% and 43% killed respectively (Figure 5F; for wild-type the numbers killed are 58% and 11%). *jun-1(gk551)* animals are not killed by SLO N402, indicating that killing by SLO in *jun-1* mutant animals is dependent upon pore formation (Figure 5F). Thus, *jun-1* is required for *C. elegans* protection against both large- and small-pore PFTs. Since KGB-1, as the master regulator of PFT defenses, controls the induction of p38-dependent sub-pathways (*ttm-1*, *ttm-2*, *hsp-4*, and *xbp-1*) and p38-independent sub-pathways (*kin-18*, *Y54E5A.1*, and *F42C5.10*), we further examined whether JUN-1 being the downstream of KGB-1 contributes to these gene regulation. The qRT-PCR analysis in wild-type versus *jun-1(gk551)* mutant animals indicated that *jun-1* is required for the Cry5B-triggered *ttm-2* and *hsp-4* but not *ttm-1*, *kin-18*, *Y54E5A.1*, and *F42C5.10* induction (Figure 5G). Consistently, we found that increased splicing (activation) of *xbp-1* in response to Cry5B does not occur in *jun-1(gk551)* mutant animals (Figure 5G). These results indicate that KGB-1-regulated genes could be further parsed to be either JUN-1 dependent or independent.

**Figure 6:**
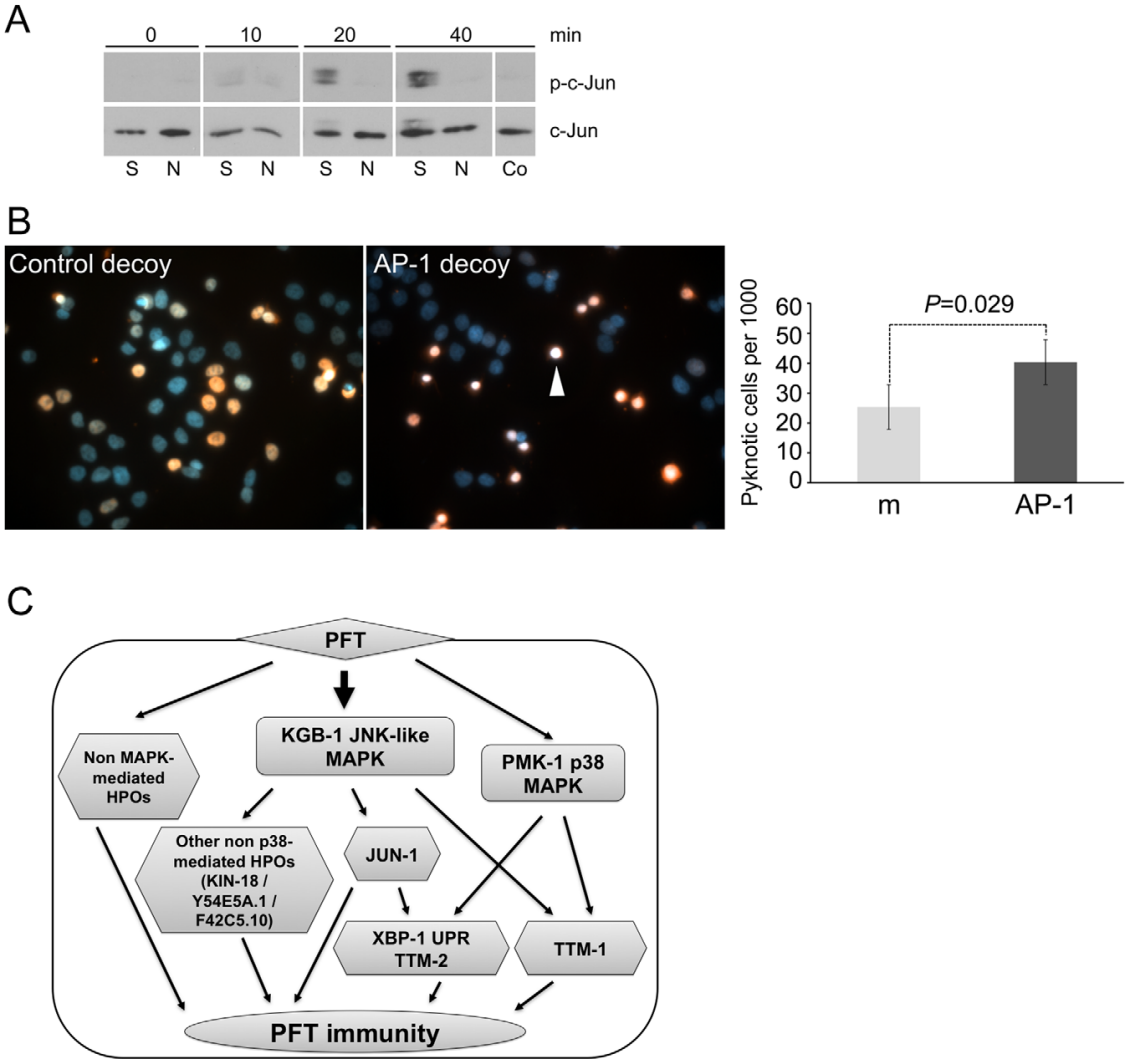
AP-1 protects against mammalian PFTs. (**A**) Western blot of mammalian c-JUN in HaCaT cells treated with 125 ng/mL SLO for time indicated. Abbreviations: S: constitutively active version of SLO, N: non-lytic mutant N402 SLO; Co: no-SLO control. (**B**) HaCaT cells were treated with SLO and biotinylated AP-1 decoy-oligonucleotide (right panel) or biotinylated control oligonucleotide (left panel). Oligonucleotide is stained red (SA-Alexa594), and DNA is stained blue (4',6-diamidino-2-phenylindole/DAPI). The images presented are a single representative from three independent assays. Cells treated with AP-1 decoy show a greater proportion of intoxicated cells with condensed chromosomes, characteristic of becoming pyknotic (*e.g.*, white arrowhead). The proportion of pyknotic cells was quantified as described in the Materials and Methods section; the bar graph shows mean values from three independent experiments (m = mismatched oligonucleotide; AP-1 = AP-1 decoy; error bars: standard error of the mean, *P* value was determined with paired, one-tailed student's t-test). (**C**) A model for interconnected regulation of defense to PFT. The KGB-1 JNK-like MAPK regulates both p38 MAPK–dependent (e.g., *ttm-1*, *ttm-2*, UPR) and p38 MAPK-independent (*e.g.*, *jun-1*, *kin-18*) PFT-induced protection genes. There are also PFT protection genes that, to date, have not been linked to either MAPK pathway.

We then considered whether our finding that AP-1 is important for PFT responses in *C. elegans* extends to mammalian cells. Using commercially available antibodies, we found that 125 ng/mL of lytic, wild-type SLO induces activation (phosphorylation) of c-JUN in HaCaT cells and that the non-lytic SLO mutant does not (Figure 6A). To test whether AP-1 is functionally important for survival of SLO-treated mammalian cells, we employed a transcription factor decoy approach [45]. HaCaT cells were simultaneously exposed to SLO and biotinylated double-stranded deoxyoligonucleotides comprising a consensus AP-1 binding site (decoy oligonucleotide), which competes with AP-1 sites of genomic promoters for binding of the transcription factor. A mismatched oligonucleotide, which does not bind AP-1, served as a control. The AP-1 decoy oligonucleotide specifically increased the proportion of pyknotic nuclei (Figure 6B). The combination of activation and decoy oligonucleotide data supports the notion that AP-1 is protective against PFT attack in mammalian cells, as it is in *C. elegans*.

## Discussion

We present the first genome-wide study of functional cellular responses to a PFT. We identify 106 *hpo* genes important for cellular protection against an attack by a small-pore PFT, Cry5B. Interactome network analysis of these genes emphasizes the importance of two MAPK pathways, p38 and JNK, in coordinating this protection. The stringency of our experimental design led us to all previously known genes that mutate to a strong Hpo phenotype and none that mutate to a weak to moderate Hpo phenotype. All of the genes on this list except two are previously uncharacterized with regards to PFT defenses. A comparison of the genes induced by PFT with these 106 important for protection against PFT revealed a statistically significant enrichment of transcriptionally-induced, but not transcriptionally-repressed, genes on the list of PFT-protective genes. These data indicate that transcriptional induction is important for PFT protection.

Our findings demonstrate that >0.5% of genes in the *C. elegans* genome play a major role in protection against pore-forming toxins. This number is large by any standard for a single cellular process. Given the likely parallels between cellular responses to pore-forming toxins and membrane ruptures that occur to cells during their normal existence, these data suggest that cells devote such a large portion of their genome to protecting against membrane holes and that this list of genes represents a rich starting point for understanding how cells cope with PFT attack and membrane damage. It is interesting to note that plasma membrane pore-formation has been suggested to play a significant role in neurological diseases of ageing, such as Alzheimer's or Parkinson's disease [10]. The significant overlap of the 106 *hpo* PFT protection genes with genes involved in promoting long life-span, suggest indeed that small ruptures of the plasma membrane play a significant role in the aging process. The fact that JNK and AP-1 are important for protection against both large-pore and small-pore PFTs indicates that at least some of the pathways cells use to deal with pores of various sizes are conserved, in contrast to previous suggestions [10], [13].

Based on interactome analyses from our genome-wide RNAi screen, we examined the requirement of the JNK MAPK pathway for PFT defenses. JNK MAPK has not been studied in any detail for its role in PFT defenses. Indeed, to date the only signal transduction pathway studied in detail has been the p38 MAPK pathway. Our study of the JNK MAPK pathway identifies it, and not p38, as the first master regulator of PFT transcriptionally-induced cellular protection. This conclusion is based on the following observations and results: 1) the JNK MAPK pathway is induced in mammalian and *C. elegans* cells by small-pore and large-pore PFTs (this study and others [38], [39]; 2) more than 50% of all genes transcriptionally induced by PFT depend upon the JNK pathway for their induction (this study); 3) 85% of the p38-dependent PFT-induced transcriptional response is controlled by JNK MAPK (this study); 4) all three of the known p38-dependent PFT-induced functional responses are dependent upon JNK (this study); 5) 50% of the *hpo* genes that mutate to a strong Hpo phenotype and that are induced by PFT are dependent upon JNK; and 6) JNK protects against both large and small-pore PFTs in *C. elegans*, the first such gene so identified. Our analyses also identify the first known downstream targets of the JNK MAPK-regulated PFT protection pathway, namely, *kin-18*, *jun-1*, *Y54E5A.1*, and *F42C5.10*. While JNK MAPK is the master regulator of induced responses, our Western blotting data suggest that JNK is not hierarchically upstream of p38 MAPK activation in response to PFT. Based on our findings we propose a model whereby the JNK pathway converges in parallel on p38-regulated PFT responsive genes to regulate the transcriptionally-induced defense response to Cry5B (Figure 6C).

Our genome-wide analyses also led to the discovery of AP-1, a key regulator of mammalian innate immunity downstream of JNK and the toll-like receptor (TLR) pathway, as important for defense against small- and large-pore PFTs in *C. elegans* and against a large-pore PFT in mammalian cells. This study is the first to identify AP-1 as involved in immunity in *C. elegans*. We also demonstrated that the UPR and *ttm-2* Cry5B-induced defense sub-pathways, but not the *ttm-1*, *kin-18*, *Y54E5A.1*, and *F42C5.10* Cry5B-induced defense sub-pathway, are transcriptionally regulated via JUN-1. Thus, AP-1 mediates some, but not all, of JNK-regulated PFT defenses (Figure 6C). As indicated by our data and reflected in our model, regulation of transcriptionally-induced cellular defenses against PFTs involves an intricately connected network of sub-pathways, with the JNK pathway hierarchically at the top of known regulators.

AP-1 is the first transcription factor to be shown to be broadly required for metazoan cells to defend against PFTs. Since the TLR does not appear to be important for *C. elegans* immunity [46], our data suggest that the role of the JNK-AP-1 pathway in protection against PFTs is one of its most ancestral functions, predating that of its role in TLR-mediated immunity.

In summary, we report the first large-scale functional study of genes involved in protecting against a small-pore PFTs, place JNK as the master regulator of known cellular defenses against small- and large-pore PFTs, and identify AP-1 as an ancient factor conserved from worms to humans involved in PFT defenses. This study demonstrates the power of integrated genome-scale approaches *in vivo* to provide vital insights and significant breakthroughs into what has been to date a daunting scientific challenge, namely understanding defenses against breaches of plasma membrane integrity.

## Materials and Methods

### *C. elegans* Strains and Microscopy

Strains were maintained at 20°C as described [47], except strains containing *glp-4(bn2)*, which were maintained at 15°C. Images for the genome-wide RNAi screen were acquired using an Olympus SZ60 dissecting microscope. Strain VP303 *rde-1(ne219); kbEx200 [rol-6(su1006);pnhx-2::RDE-1]* was provided by Kevin Strange (Vanderbilt University) [48]. *Phsp-4*::GFP and *Phsp-4*::GFP;*kgb-1(um3)*) animals were placed on 2% agarose pads containing 0.1% sodium azide and were imaged on an Olympus BX60 microscope with a 10x objective.

### Toxin Preparation

Purified Cry5B was prepared as described [49] and suspended either in water for the genome-wide RNAi screen, or dissolved in 20 mM HEPES (pH 8.0) for liquid mortality assays. *E. coli*-expressed Cry5B was prepared as described previously [50], except the overnight culture was 5 times diluted before IPTG induction in OP50 expression system. For the microarray experiment, conditions were carried out as described [11].

### Cry5B Activation and Ion Channel Currents Recording in Planar Lipid Bilayers

Solubilized Cry5B protoxin was incubated at 30°C for two weeks, allowing co-purifying native Bt protease(s) to activate it into a 59 kDa fragment. The 59 kDa Cry5B was purified by gel filtration (Superdex 75 16/60). Ionic currents passing through activated Cry5B proteins (5-10 µg/mL) reconstituted into planar lipid bilayers were recorded at various holding voltages applied to phospholipid membranes (phosphatidylethanolamine∶phosphatidylcholine∶cholesterol (7∶2∶1 w/w)) painted across a 250-µm hole drilled in a Teflon wall separating two 600-µl chambers filled with 150 mM KCl, 10 mM Tris, pH 9.0. Data were recorded with an Axopatch-1D amplifier (Axon Instruments, Foster City, CA), filtered at 600Hz, digitized and analyzed using pClamp6 software (Axon Instruments) [51].

### Genome-Wide RNAi Screen and Liquid RNAi Assays

RNAi feeding for the genome-wide screen was performed in a 24-well format modified from a published protocol [52]. Bacteria containing each RNAi clone were cultured in 100 µL Luria Broth media containing 50 µg/mL carbenicillin in a 96-well format for 16–18 hrs. 30 µl of each culture was seeded in one well of a 24-well plate containing NGM agar, 1 mM IPTG and 50 µg/ml carbenicillin (each plate was set up in duplicate). For each batch of RNAi clones tested, empty vector (L4440) and *tir-1* RNAi clones were included as negative (no knock-down) and positive (Hpo) controls respectively. After overnight incubation, ∼20 synchronized RNAi-sensitive *rrf-3(pk1426)* L1 worms were placed in each well and allowed to develop to L4 stage at 20°C. 30 µL of total 1 µg purified Cry5B resuspended in water was added to RNAi-fed worms in each well (*rrf-3* mutants have a normal response to Cry5B [11]). The plates were covered with porous Rayon films (VWR, West Chester, PA) to allow air exchange. Hypersensitivity to PFT relative to no knock-down (empty vector) controls was assessed after 48 hours by light phase microscopy. The initial Hpo hits were individually retested in duplicate under the same conditions (second round). In addition, for each RNAi clone, a single well was set up and treated with water alone (no toxin) to assay for ill health in the absence of toxin. To be considered a *hpo* gene, we looked for lack of ill health and developmental problems in the no-toxin well and either >50% moderately Hpo animals in both wells or >33% severely Hpo animals in both wells. Two hundred and sixty nine such genes were found in total. For the quantitative validation test (third round), bacteria from these RNAi hits were cultured overnight, induced with 1mM IPTG for 1 hour at 37°C, and resuspended in S-media. We then added ∼20 *glp-4(bn2)*;*rrf-3(pk1426)* L1 animals to each well of a 48-well plate with RNAi bacteria (triplicate wells for each clone). The plates were gently rocked at 25°C for ∼30 hours until the animals had developed to the L4 stage. Then we added 10 µg/mL Cry5B or 20 mM HEPES pH 8.0 control into each well and quantitated the percentage of worms that were killed after six days at 25°C. The worm viability was determined based on movement and worms failed to respond to several gentle touches with a platinum pick were scored as dead. The survival on each RNAi treatment was normalized to L4440 treatment in the same batch, to help account for differences in toxicity from batch to batch of the experiment. We sequenced the 106 *hpo* gene clones to confirm their identities. The quadruplicate repeat triple-well verification was performed with the similar procedures except that no plate rocking was performed and data were analyzed via analysis of variance (ANOVA) and Dunnett's post-test. Statistical data analyses here and elsewhere were performed using Prism 5.0 (GraphPad, San Diego, CA).

For intestinal-specific liquid RNAi, bacteria for *kgb-1* RNAi or L4440 empty vector were first cultured overnight, induced with 1mM IPTG for 1 hour at 37°C, and resuspended in S-media. Approximately 20 synchronized L1 stage VP303 worms were fed with *kgb-1* or L4440 RNAi in 48 wells plates, grown to the L4 stage in ∼40 hours at 25°C and FUdR was then added to a final concentration of 200 µM. Then Cry5B to a final concentration of 10 µg/mL or 20 mM HEPES pH 8.0 control were added into each well and the percentage of worms that were killed after six days at 25°C was scored. The comparison of survivals between *kgb-1* RNAi and empty control was done with one-tailed Student's t-test.

### Microarrays and Bioinformatics

The RNA samples were collected from three independent experiments as described [11]. *glp-4(bn2)* animals lack a functional germline and have otherwise normal response to Cry5B [11]. Use of these animals removes a major tissue from the animals, allowing for intestinal mRNAs to represent a larger portion of the total RNA population. Raw microarray datasets were normalized using Bioconductor's [53] Robust Multi-array Analysis (RMA) [54], [55] in R language. Linear Models for Microarray Data (LIMMA) [56] was used to determine a set differentially expressed genes. The cutoff p-value used was 0.01 with minimum 1.5 or 2 fold change. The data were clustered to reveal prominent groups of transcripts with similar changes in expression pattern using a κ-means (κ = 4) algorithm.

The gene enrichment analysis for microarray was done with DAVID PANTHER annotation tool [57]. Only enriched categories with three or more counts and *P*<0.05 were kept. The statistical significance of the observed overlap between different gene lists (*e.g.*, genome-wide RNAi screens, *hpo* vs. induced genes) was calculated by a modified Fisher's exact test [58]. The PFT immunity interaction network was generated by combining data from our genome-wide RNAi screen and a modified *C. elegans* integrated functional network (WI8∪Literature∪Interolog∪Genetic) database with several manually curated interactions [34]. Interactions were only maintained if at least one of the nodes was *hpo*. The resulting networks were visualized using Cytoscape 2.6.3 [59].

### Quantitative RT-PCR

Total RNA was isolated from approximately 5,000 L4 worms and cDNA was made from 2 µg of total RNA with MoMLV-reverse transcriptase (Promega, Madison, WI) and oligo-dT primers for 90 minutes at 42°C in 20 µl reaction mixtures. qRT-PCR (triplicate independent experiments, normalization to *eft-2*) was then carried out as described [14]. Primers for qRT-PCR are listed in Table S3 except for *eft-2*, *ttm-2*, *hsp-4*, and *xbp-1* spliced form, which have been described before [14], [15]. Transcript levels among mutants and wild-type animals for a given gene were compared using one-way ANOVA with Tukey's HSD post hoc test or one-tailed Student's t-test (Figure 3A and 5G only).

### KGB-1 Immunoblotting

Approximately 750 L1 stage worms were grown in a single well of a 48 well plate, treated with indicated concentrations of Cry5B and prepared as described before for phospho p38 MAPK and α-tubulin [14]. The same PVDF membranes were stripped with Restore Western Blot Stripping Buffer (Thermo Fisher Scientific Inc., Waltham, MA) and reprobed as needed. The probing order is phospho-KGB-1 (1∶1000) first, phospho-p38 MAPK and α-tubulin second, and total KGB-1 antiserum (1∶5000) third.

### *C. elegans* Mortality Assays with Mutants

Cry5B mortality assays with wild-type animals and *kgb-1* and *sek-1* mutants were carried out for 8 days at 20°C in three independent experiments as per standard protocol[50]. LC_50_ values and confidence intervals were calculated as described [60]. For SLO and N402 assays, synchronized L4 worms were prepared in complete or calcium-free S-medium with 50 µg/mL of toxin dissolved in phosphate buffered saline (PBS; PBS alone was used in negative controls). The survival of animals was scored after six days at 25°C and differences analyzed by analyzed with one-tailed Student's t-test. The *P. aeruginosa* PA14 survival assay was performed on slow-killing plates as described [14]. For the heat stress analysis, synchronized N2 and *jun-1(gk551)* animals were shifted to 35°C as two-day-old adults and survival was scored every two hours after the treatment as described [61]. Three lifespan assays per worm strain were done, and all show the same trends. The data presented in the paper are from a representative experiment. Data are plotted as a Kaplan–Meier survival plot and analyzed for significance using the Log Rank test.

### Preparation of SLO and Mammalian Cell Experiments

Recombinant streptolysin O and non-lytic mutant N402 were prepared as described previously [43], [62]. Growth of the human keratinocyte cell line HaCaT has been previously described [63]. Cells were seeded at a density of 0.5x10^6^ cells in 12-well plates, cultured overnight and treated with 125 ng/ml SLO or non-lytic mutant N402 for indicated times. After washing with PBS, cells were lysed by addition of 50 µl 2xSDS-loading buffer. Proteins were separated by SDS-PAGE and electroblotted onto nitrocellulose membrane, incubated with rabbit anti-phospho Ser63-c-jun (1∶1000; Cell Signaling Technology) over night at 4°C, washed, and incubated with goat anti-rabbit IgG HRP-conjugate (1∶2000; New England Biolabs). After three washes, bound antibody was detected by ECL. The same membrane was stripped with TBS containing 2% SDS and 100 mM β-mercaptoethanol for 50 min at 50°C and reprobed with rabbit anti-c-jun (1∶1000; Cell Signaling Technology).

For the AP-1 decoy experiment, HaCaT cells were treated in Ca^2+-^free medium with 500 ng/mL SLO and 3 µM biotinylated AP-1 decoy oligodeoxynucleotide (ODN), or mismatched AP-1 decoy ODN for 30 min, (Genedetect, Auckland, New Zealand). Subsequently, cells were washed twice with PBS and incubated in full culture medium. After incubation for 4 hours, cells were fixed with 4% paraformaldehyde, permeabilized with 0.1% triton-X100. Biotinylated ODN were stained with AlexaFluor594-conjugated streptavidin; nuclei were visualized with DAPI stain (10 µg/mL). After washing, cells were examined by fluorescence microscopy using an Axiovert 200M microscope (Zeiss, Germany). The relative intensity of DAPI staining was quantified for ca. 800 cells per treatment and experiment, applying the analysis tool of Photoshop to digital images in RGB-mode. Nuclei were considered pyknotic if they were condensed to a diameter<50% of average and showed more intense DAPI uptake (>2-fold of average intensity)[64].

## Supporting Information

Figure S1 Functional classification of *hpo* genes based on Wormbase annotation.
(0.11 MB TIF)

Figure S2 A qualitative analysis of the sensitivity of worms with *mek-1(ks54)* loss of function mutation. Animals were exposed for 72 hours to OP50 *E. coli* expressing empty vector (no toxin) or diluted Cry5B PFT (10%). *mek-1* mutant animals are Hpo as they are significantly more intoxicated by Cry5B PFT than wild-type animals.
(0.65 MB TIF)

Figure S3 *jun-1* loss-of-function increases resistance of worms to heat stress. A and B represent the other two repeats of the heat stress assays.
(0.16 MB TIF)

Figure S4 *jun-1* loss-of-function increases resistance of worms to PA14 infection. A and B represent the other two repeats of the PA14 life-span assays.
(0.17 MB TIF)

Table S1 Further confirmation of a subset of Hpo RNAi clones.
(0.03 MB XLS)

Table S2 Statistics for stress response assays.
(0.03 MB XLS)

Table S3 Primers sequences for qRT-PCR.
(0.03 MB XLS)

Supporting Information S1 Microarray data. Data from microarray experiments showing all genes 2-fold up and down following PFT treatment and their dependence upon MAPK pathways and all genes 1.5-fold up and down following PFT treatment.
(0.87 MB XLS)
