# Supplementary figures and images for: Global Functional Analyses of Cellular Responses to Pore-Forming Toxins
Source: PLoS Pathog. 2011 Mar 3;7(3):e1001314. doi: 10.1371/journal.ppat.1001314 (PMC3048360; doi:10.1371/journal.ppat.1001314)

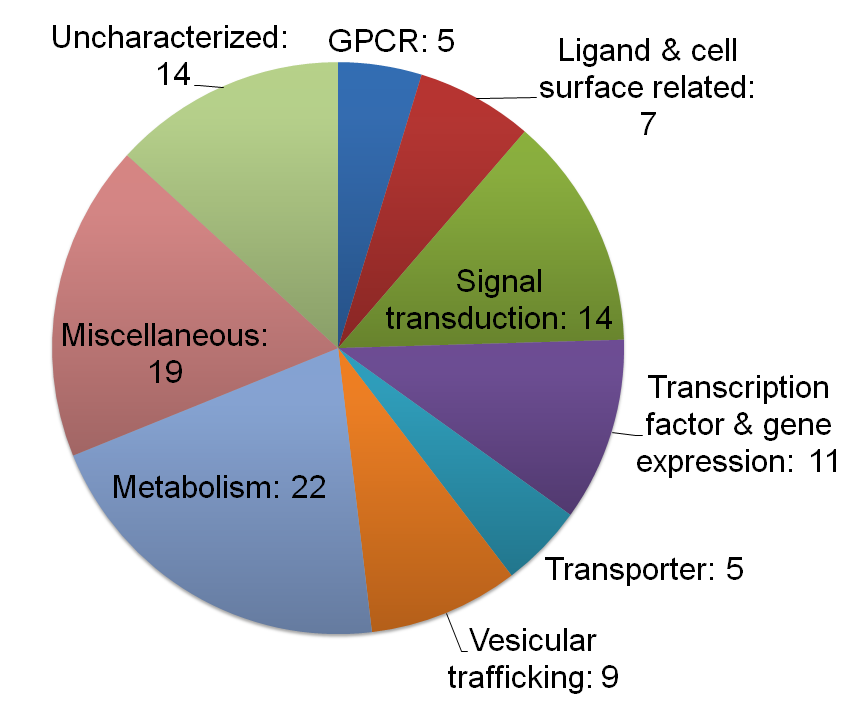

Supplement: Figure S1 — Functional classification of hpo genes based on Wormbase annotation. (0.11 MB TIF) [file ppat.1001314.s001.tif]

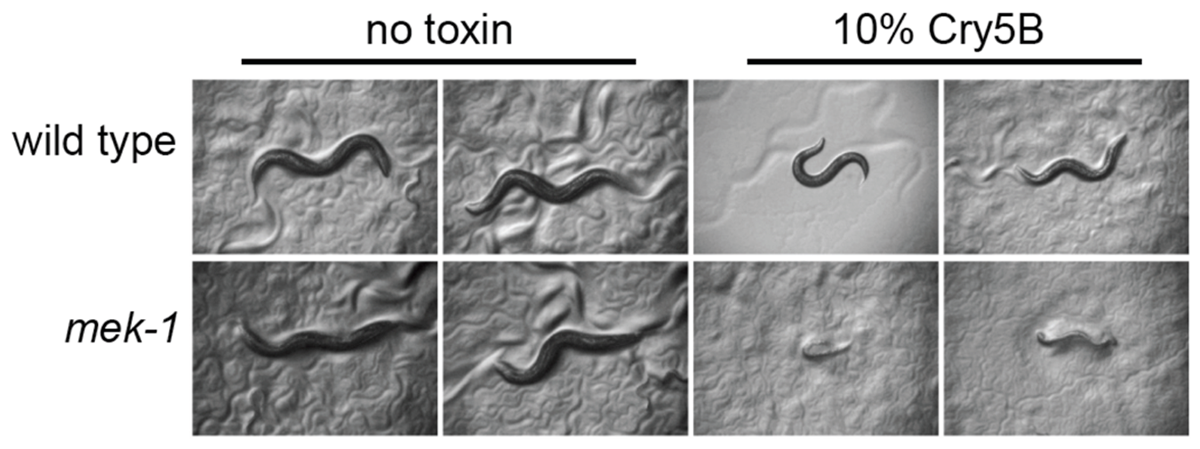

Supplement: Figure S2 — A qualitative analysis of the sensitivity of worms with mek-1(ks54) loss of function mutation. Animals were exposed for 72 hours to OP50 E. coli expressing empty vector (no toxin) or diluted Cry5B PFT (10%). mek-1 mutant animals are Hpo as they are significantly more intoxicated by Cry5B PFT than wild-type animals. (0.65 MB TIF) [file ppat.1001314.s002.tif]

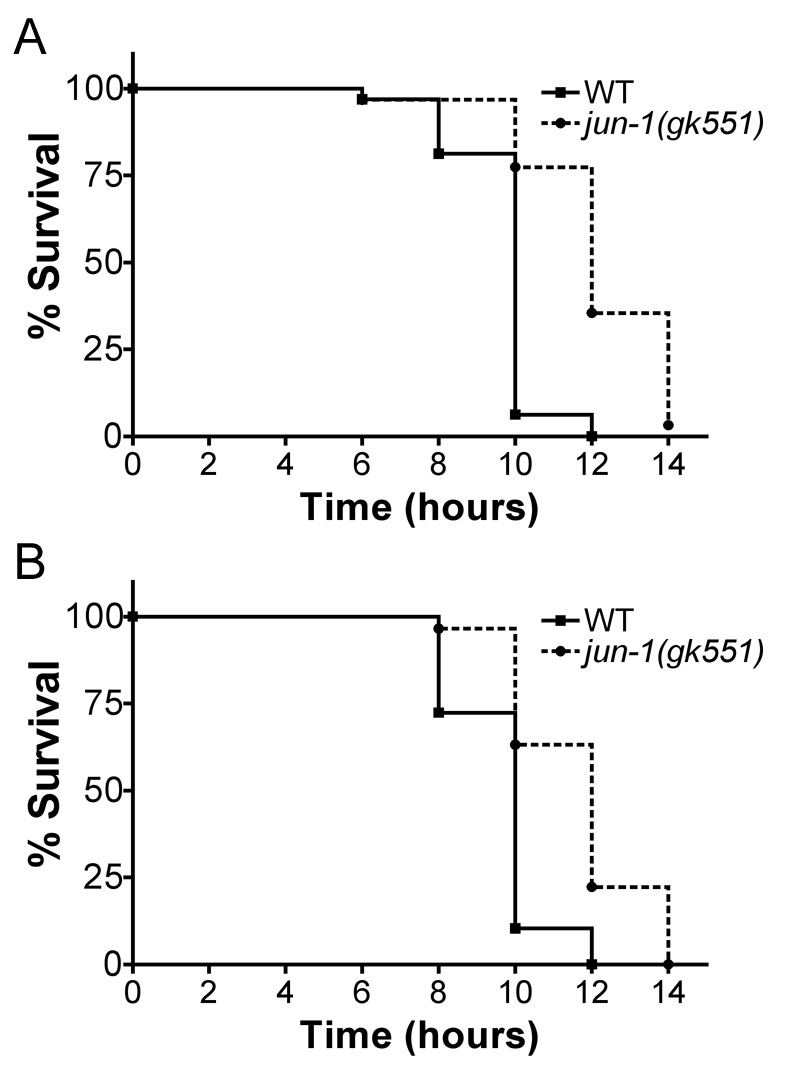

Supplement: Figure S3 — jun-1 loss-of-function increases resistance of worms to heat stress. A and B represent the other two repeats of the heat stress assays. (0.16 MB TIF) [file ppat.1001314.s003.tif]

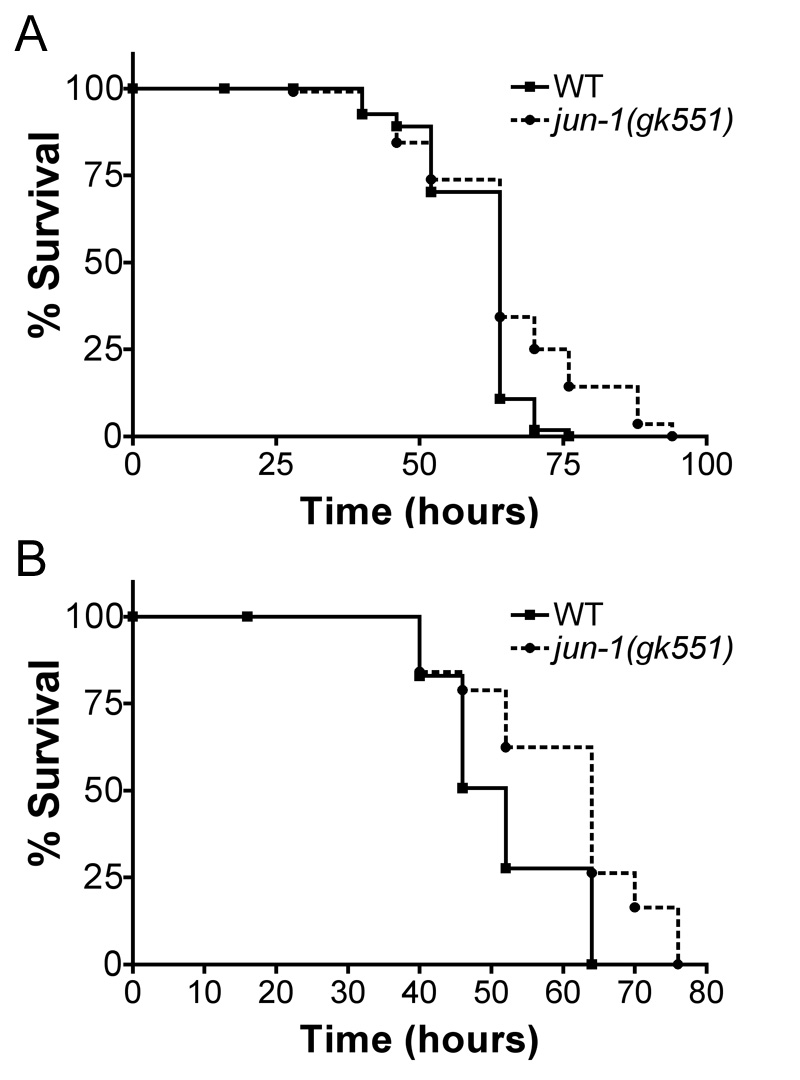

Supplement: Figure S4 — jun-1 loss-of-function increases resistance of worms to PA14 infection. A and B represent the other two repeats of the PA14 life-span assays. (0.17 MB TIF) [file ppat.1001314.s004.tif]
